# Polypeptides Targeting *Paracoccidioides brasiliensis* Drk1

**DOI:** 10.3390/jof9100980

**Published:** 2023-09-29

**Authors:** Caroline Maria Marcos, Haroldo Cesar de Oliveira, Patricia Akemi Assato, Lariane Teodoro de Oliveira, Nathália Fregonezi, Kelvin Sousa dos Santos, Caroline Barcelos Costa-Orlandi, Ana Marisa Fusco-Almeida, Maria José Soares Mendes-Giannini

**Affiliations:** 1 School of Pharmaceutical Sciences, São Paulo State University (UNESP), Araraquara 14800-903, Brazil; marcos_caroline@yahoo.com.br (C.M.M.); haroldocdoliveira@gmail.com (H.C.d.O.); patricia.assato@gmail.com (P.A.A.); lariane.t@hotmail.com (L.T.d.O.); nathfregonezi@gmail.com (N.F.); k.santos@unesp.br (K.S.d.S.); carolbarceloscosta@gmail.com (C.B.C.-O.); ana.marisa@unesp.br (A.M.F.-A.); 2 Instituto Carlos Chagas, Fundação Oswaldo Cruz (Fiocruz), Curitiba 81350-010, Brazil; 3 Laboratório Central de Multiusuários, Faculdade de Ciências Agronômicas, Campus Botucatu, UNESP—Universidade Estadual Paulista, São Paulo 18610-034, Brazil

**Keywords:** *Paracoccidioides brasiliensis*, Drk1, phage-display, peptides

## Abstract

Considering the toxicity of conventional therapeutic approaches and the importance of precise mechanistic targets, it is important to explore signaling pathways implicated in fungal pathobiology. Moreover, treatment of paracoccidioidomycosis, a systemic mycosis caused by a dimorphic fungus, requires prolonged therapeutic regimens. Among the numerous factors underpinning the establishment of *Paracoccidioides* spp. infection, the capacity to transition from the mycelial to the yeast form is of pivotal importance. The Drk1 protein of *Paracoccidioides brasiliensis* likely plays a decisive role in this morphological shift and subsequent virulence. We identified peptides with affinity for the PbDrk1 protein using the phage-display method and assessed the effects of these peptides on *P. brasiliensis*. The peptides were found to inhibit the phase transition of *P. brasiliensis.* Furthermore, a substantial proportion of these peptides prevented adhesion to pneumocytes. Although these peptides may not possess inherent antifungal properties, they can augment the effects of certain antifungal agents. Notably, the cell wall architecture of *P. brasiliensis* appears to be modulated by peptide intervention, resulting in a reduced abundance of glycosylated proteins and lipids. These peptides were also evaluated for their efficacy in a *Galleria mellonella* model and shown to contribute to enhanced larval survival rates. The role of PbDrk1, which is notably absent in mammals, should be further investigated to improve the understanding of its functional role in *P. brasiliensis*, which may be helpful for designing novel therapeutic modalities.

## 1. Introduction

Antifungal therapeutics exhibit limited efficacy in treating fungal infections, partially because of the constrained spectrum of activity exhibited by conventional drugs, compounded by the lack of dedicated public policies addressing such infections [1,2]. Pathogenic fungi have gained increased attention in recent decades because of a surge in the number of mycosis cases. This escalation is closely associated with the expanding population of susceptible individuals, particularly patients who are immunocompromised, from organ transplant recipients to those undergoing autoimmune disease treatments, and includes those afflicted by immunosuppressive conditions, such as HIV infection and AIDS. Notably, the mortality toll of fungal infections is nearly as high the global burden of malaria and tuberculosis [3].

Furthermore, the emergence of resistance to selected antifungal agents underscores the need for innovative antifungal compounds with diverse targets. *Paracoccidioides* spp. are thermodimorphic fungi responsible for causing paracoccidioidomycosis (PCM). This systemic, chronic, and progressive mycosis is prevalent and endemic in Latin America and is typically contracted through inhalation of conidia that subsequently transform into a pathogenic yeast form upon reaching the lungs [4,5].

Therapeutic management of PCM requires prolonged treatment periods, often involving agents such as amphotericin B (AMB), itraconazole (Itra), and cotrimoxazole. However, these interventions are associated with adverse events [6]. Currently, the main antifungal targets include ergosterol (targeted by the polyene class), sterol 14 demethylase (inhibited by azoles), β-glucan synthesis (affected by echinocandins), and interference with RNA and DNA synthesis (achieved using pyrimidine analogs). However, each approach has inherent limitations, including elevated toxicity profiles and susceptibility to antifungal resistance [2,7]. Thus, novel strategies are being explored, including drug repurposing initiatives [8] and natural or synthetic compounds tailored to targets encompassing distinct facets of the cell wall and membrane architecture, metabolic pathways, signal transduction cascades, gene expression modulation, and virulence factors [9,10,11,12].

A prerequisite for exploring therapeutic avenues is a comprehensive understanding of the intricate pathogenesis of fungal infections. Infection involves an interplay between pathogens and hosts, which is contingent on fungal virulence and individual susceptibility. Within *Paracoccidioides brasiliensis*, an array of virulence factors has been documented [13]. Notably, a recently highlighted factor, dimorphism-regulating histidine kinase 1 (Drk1), was predicted as an important target for inhibitory evaluation, as previously demonstrated to other dimorphic fungi [14,15,16,17] and *P. brasiliensis* [18,19,20].

As a variant of the two-component signal transduction system, Drk1 possesses catalytic and receiver domains [21,22]. This protein is an integral constituent of the high-osmolarity glycerol (HOG pathway) that underpins cellular adaptation to diverse stressors. This system encompasses a sensor-effector network that orchestrates cellular responses under changing conditions [23,24,25]. By initiating consecutive phosphorylation events in mitogen-activated protein kinases (MAPK), MAPKKK, MAPKK, and Hog1 MAPK, phosphorylated Hog1 MAPK relocates to the nucleus where it is involved in transcriptional and post-transcriptional regulation [26]. Each cue is sensed and transmitted to the HOG pathway, mostly a two-component signal transduction system as Drk1, because of the phosphorylation of histidine and its transference to aspartate residues [27]

It can trigger consecutive phosphorylation reactions in mitogen-activated protein kinases (MAPK), MAPKKK, MAPKK, and Hog1 MAPK, the latter when phosphorylated is translocated to the nucleus regulating the transcription and post-transcription [26]. Each cue is sensed and transmitted to the HOG pathway, mostly a two-component signal transduction system as Drk1, because of the phosphorylation of histidine and its transference to aspartate residues [27].

Although the role of the HOG pathway in *P. brasiliensis* adaptation is not well understood, there are indications of its importance in multiple aspects. The mycelium-to-yeast transition is facilitated by Drk1, a group III histidine kinase. Interestingly, the expression of DRK1 is increased during the yeast phase, particularly in more virulent isolates and in response to osmotic conditions, as determined using real-time PCR [19] and functional suppression of DRK1, which yields transformative phenotypic alterations in *P. brasiliensis*. Notable changes include elongated cellular morphology at 37 °C, attenuated virulence in a *Galleria mellonella* model, diminished chitin content, augmented resistance to osmotic and cell wall stresses, and caspofungin resistance coupled with heightened sensitivity to Itra [18]. Research by Navarro et al. [20] involving fludioxonil, another group III histidine kinase inhibitor, revealed the roles of Drk1 in pathogen resistance to cell-wall-disrupting agents, modulation of cell-wall-related genes, increased macrophage phagocytosis, and β-glucan production.

However, the mechanisms of fludioxonil’s impact are unclear. A direct interaction was suggested between this fungicide and the fungal cell protein hybrid kinase (HHK). However, studies have revealed that the interactions are complex and that the effects of fludioxonil do not stem from a direct interaction with HHK. Instead, the fungicide likely induces oxidative stress, which is a perturbation detected by HHK. However, oxidative stress alone is insufficient to induce lethality in fungal cells. Studies are needed to determine the mechanisms governing the effects of fludioxonil and its mode of action [28].

Given the multifaceted roles attributed to *P. brasiliensis* Drk1, which is thought to have a critical role in physiological processes, virulence, dimorphism, and cell wall remodeling, we used a phage display technique to identify peptides capable of interacting with Drk1. We also examined the effects of these interactions on *P. brasiliensis* host interplay and physiological dynamics. This study demonstrates the intricacies of Drk1’s functional repertoire and provides insight into the broader landscape of fungal pathogenesis and potential avenues for therapeutic interventions.

## 2. Materials and Methods

### 2.1. Fungal Isolates and Growth Conditions

The *P. brasiliensis* strain Pb18 was cultivated on solid Fava-Netto medium [29] for 5 days at 37 °C and then subcultured for 72 h in liquid brain–heart infusion (BHI) medium supplemented with 1% glucose at 37 °C. Cultivation was performed with continuous agitation at 150 rpm, and the resulting culture was used for assays. Cell viability was assessed using the trypan blue exclusion test [30].

### 2.2. Phage-Display Library and Biopanning

The M13KE phage vector from the Ph.D.TM—7 Phage Display Peptide Library (New England Biolabs, Ipswich, MA, USA) was used for biopanning. This library contains randomized heptapeptides fused to the phage minor coat protein pIII N-terminus. Biopanning was performed to isolate high-affinity phages capable of binding to the recombinant Drk1 protein of *P. brasiliensis* previously obtained by Marcos et al. [18].

A 96-well microtiter plate was coated with recombinant PbDrk1 protein at 100 µg/mL in 0.1 M NaHCO_3_ (pH 8.6) overnight at 4 °C in a humidified chamber. Excess unbound protein solution was removed, and the plate wells were blocked using 5 mg/mL bovine serum albumin (BSA) in 0.1 M NaHCO_3_ for 1 h at 4 °C. The wells of the plate were thoroughly washed six times with 0.1% Tween-20 in Tris-buffered saline [TBS]) (50 mM Tris, pH 7.5, and 150 mM NaCl; TBS-T).

The original phage library was diluted 100-fold in TBS-T, and 100 µL of the diluted library was added to the plate wells. This step was performed at room temperature under gentle agitation for 1 h. Unbound phages were subsequently washed away by 10 rounds of washing with TBS-T. Bound phages were then eluted from the plate wells using 100 µL of a solution containing 0.2 M glycine-HCl (pH 2.2) and 1 mg/mL BSA for 10 min at room temperature with gentle agitation. The eluted phages were neutralized using 15 µL of 1 M Tris-HCl (pH 9.1).

A small aliquot (1 µL) of the eluted phages was titrated, whereas the remaining volume was amplified to attain sufficient copies for subsequent rounds of biopanning.

### 2.3. Phage Amplification

The amplified eluted phages were individually propagated by infecting 20 mL *Escherichia coli* K12 ER2738 culture. The bacterial culture, previously grown in Luria–Bertani (LB) medium supplemented with 20 µg/mL tetracycline, was grown until the early log phase at 37 °C with agitation at 250 rpm. The bacterial cells were removed by centrifugation at 12,000× *g* for 10 min. The supernatant was collected and subjected to a second round of centrifugation. Approximately 80% of the upper portion was purified via precipitation using 20% polyethylene glycol 8000 (PEG) and 2.5 M NaCl (PEG/NaCl). After further centrifugation, the phage pellet was resuspended in 1 mL TBS (50 mM Tris-HCl, pH 7.5) containing 150 mM NaCl. The mixture was reprecipitated with PEG/NaCl for 1 h on ice. The phage pellet was centrifuged again and resuspended in 200 µL of TBS.

The amplified phage was used for subsequent rounds of biopanning (four rounds), as previously described (with TBS solution containing an increased Tween-20 concentration of 0.5%). An additional biopanning round was conducted using phage clones exhibiting a higher frequency. This step was performed using a well plate lacking the Drk1 target to validate the specificity of phage binding to the PbDrk1 recombinant protein rather than to the plastic plate.

### 2.4. Phage Titration

Phage titration was conducted to assess the viral particle output. Both the nonamplified (diluted from 10^−1^ to 10^−4^ in LB medium) and amplified eluates (diluted from 10^−9^ to 10^−12^ in LB medium) were used to infect an *E. coli* K12 ER2738 culture. This bacterial culture, previously cultivated as described in Section 2.3, was brought to the mid-log phase at 37 °C with agitation at 250 rpm. The bacterial culture (200 µL) was mixed separately with 10 µL of each eluate dilution, followed by vortexing and incubation at room temperature for 5 min. The resulting suspensions were combined with 3 mL of Top-agar (1% tryptone, 0.5% yeast extract, 0.5% NaCl, and 0.7% bacteriological agar) that had been pre-warmed to 45 °C. After thorough vortexing, the mixture was immediately spread onto prewarmed LB medium plates supplemented with isopro-pyl-beta-D-thiogalactopyranoside/5-bromo-4-chloro-3-indolyl-α-D-galactoside (IPTG/X-gal). Following overnight incubation at 37 °C, the plaques were counted to determine viral particle input and output during the biopanning cycles.

### 2.5. DNA Extraction for Sequencing and Peptides Sequence Deduction

After the fourth round of biopanning, individual positive phage clones (indicated by blue plaques) were used to infect 1 mL of an *E. coli* K12 ER2738 culture grown overnight and diluted 1:100 in LB medium. Incubation was performed for 4 h at 37 °C. The cultures were centrifuged at 12,000× *g* for 30 s, and 500 µL of the supernatants was precipitated using 200 µL of PEG/NaCl at room temperature for 10–20 min with intermittent tube inversion. The samples were centrifuged at 12,000× *g* for 10 min, and DNA was extracted from the resulting phage pellet.

Single-stranded DNA was extracted from the phage pellet by combining 100 µL of iodide buffer (10 mM Tris-HCl pH 8.0, 1 mM EDTA, and 4 M NaI) with 250 µL of absolute ethanol. The mixture was incubated for 20 min at room temperature and centrifuged at 20,800× *g* for 10 min at 4 °C; the resulting pellets were washed with 70% ethanol. The pellets were dried, resuspended in TE buffer pH 8.0 (Tris/EDTA), and used for sequencing.

For the sequencing reaction, the phage DNA samples (200 ng) were combined with the 188gIII sequencing primer (5′- ATAAGTATAGCCCGGAATAGG-3′; 3.2 pmol) and the BigDye^®^ Terminator v3.1 Cycle Sequencing kit (Applied Biosystems, Foster City, CA, USA). The thermocycler program was as follows: 35 cycles at 95 °C for 20 s, 50 °C for 15 s, and 60 °C for 1 min (Veriti^TM^, Thermo Fisher Scientific, Waltham, MA, USA). The DNA was precipitated, resuspended in dilution buffer, and sequenced on an ABI PRISM 3130 (Applied Biosystems). Amino acid sequences were deduced from the phage display DNA sequences using online bioinformatics tools such as Reverse Complement (http://www.bioinformatics.org/sms/rev_comp.html; accessed on 15 October 2017) and Reverse Translate (http://www.bioinformatics.org/sms2/rev_trans.html; accessed on 15 October 2017). The four most prevalent polypeptides identified in phage clone sequencing were synthesized by Peptide 2.0 Company (Chantilly, VA, USA).

### 2.6. Reactivity of Individual Peptides with P. brasiliensis and PbDrk1 Protein Using Enzyme-Linked Immunosorbent Assay (ELISA)

We coated 96-well plates with 50 µL of peptides (10 µg/mL) in carbonate buffer (pH 9.6) for 1 h at room temperature, washed the plates with PBS, and blocked the wells with 1% BSA for 2 h at 37 °C. The plate was incubated with PbDrk1 protein (10 µg/mL) in PBS or with 100 µL of *P. brasiliensis* yeast cells (10^6^ cells/mL) for 15 h at 37 °C. The plates were washed with PBS-Tween-20 0.05% (PBS-T), after which primary antibodies (1:100, in PBS-T 0.5% BSA) were added and incubated for 1 h at 37 °C. The anti-PbDrk1 antibody [18] and anti-cell free antibody were used to detect the Drk1 recombinant protein and *P. brasiliensis* well plate, respectively. After washing, anti-rabbit IgG-HRP (1:2000, in PBS-T 0.5% BSA) was added as the secondary antibody and incubated for 1 h at 37 °C, followed by PBS washing. The colorimetric reaction developed using 0.2 M sodium phosphate, 0.1 M citric acid, 0.4 mg/mL o-phenylenediamine, and 30% hydrogen peroxide for 10 min, and the reaction was stopped by adding 3N HCl. Absorbance was measured at 490 nm [31]. As a control, the same procedure was performed without the addition of peptide.

### 2.7. Antifungal Activity of Peptides

The activity of the peptides against *P. brasiliensis* was evaluated using a microdilution microplate assay method described in the M27-A3 document (CLSI) together with AlamarBlue (DAL 1100, Life Technologies, Carlsbad, CA, USA) reagent as described by de Paula e Silva et al. [32]. AMB and Itra were used as controls, and the peptide concentrations ranged from 0.765 to 1.600 µg/mL. The peptides and control solutions were prepared in RPMI 1640 (Gibco, Grand Island, NY, USA) medium supplemented with 2% glucose. The plates were incubated for 48 h at 35 °C and 150 rpm, followed by incubation for an additional 24 h after adding Alamar-Blue and visual interpretation of the minimum inhibitory concentration.

### 2.8. Peptides-Antifungal Combinations

The potentiation effects of peptides on antifungal activity (Itra and AMB; Sigma, St. Louis, MO, USA,) was evaluated in a microplate assay using AlamarBlue (Invitrogen, Carlsbad, CA, USA) and the analysis in a spectrophotometer at 570 and 600 nm. The peptide concentration (200 µg/mL) was fixed, whereas the concentration of the antifungal was varied at a subinhibitory concentration. Inoculum, peptides, and the antifungal agent were prepared as described above.

### 2.9. Peptides Toxicity

#### 2.9.1. In Pneumocytes

Cytotoxicity was evaluated in a 96-well plate format after 24 h of exposure to 25–1.600 µg/mL peptides using the resazurin colorimetric method (Sigma-Aldrich). To each well, 10^5^ pneumocytes A549 in complete DMEM (10% SFB) were added, followed by incubation at 37 °C and 5% CO_2_ for 24 h to obtain a confluent monolayer. The medium was removed, and 100 µL of each tested dilution of the peptides in complete DMEM was added to the cell monolayer and incubated for another 24 h under the same conditions. Resazurin solution (30 µL of 0.01%) was added, and the plates were incubated for 4 h under the same conditions, followed by absorbance measurement at wavelengths of 570 and 600 nm.

#### 2.9.2. In *G. mellonella*

*Galleria mellonella* larvae (0.15–0.20 mg) were separated into groups and incubated at 37 °C in the dark without food. After cleaning the proleg area with 70% ethanol, the larvae were inoculated with 10 µL of each peptide at concentrations ranging from 50 to 100 µg/larva of each peptide in PBS via the left proleg using a Hamilton syringe. Percentage survival was monitored for 7 days by counting daily deaths (lack of physical movement). Untreated cells that were injected with the same volume of PBS were used as controls. Ten larvae were used for each condition, and the experiment was performed three times.

### 2.10. Paracoccidioides Brasiliensis-Pneumocytes and P. brasiliensis-Extracellular Matrix Adhesion after Peptides Treatment Using Enzyme-Linked Immunosorbent Assay

For the adhesion assay of extracellular matrix (ECM) components, 96-well plates were pre-sensitized with 10 µg/mL of laminin and fibronectin individually in carbonate buffer (0.05 M, pH 9.6) overnight at 4 °C and then for 1 h at room temperature. After washing with PBS-T, the plates were blocked with BSA for 2 h at 37 °C.

The inoculum containing 10^6^ yeast cells/mL of *P. brasiliensis* was incubated with 200 µg/mL of each peptide for 1 h at 37 °C with 150 rpm of agitation. The inoculum was washed with PBS and resuspended in complete DMEM medium. A group without peptide treatment was used as control. Yeast cells (100 µL) treated and not treated with each peptide were added to the plate and incubated for 15 h at 37 °C and 5% CO_2_, after which the reaction was performed as described by Oliveira et al. [31] using anti-cell free *P. brasiliensis* antibody (1:100 in PBS-T containing 0.5% BSA, 1 h at 37 °C, as a primary antibody) and anti-rabbit IgG-HRP (1:2000 in PBS-T containing 0.05% BSA, for 1 h at 37 °C) after prewashing the plate with PBS-T.

Additionally, 100 µL of *P. brasiliensis* treated and not treated with peptides was added to the plates containing ECM components and incubated for 15 h at 37 °C followed by ELISA, as described above.

After washing the plates with PBS-T, peroxidase activity was evaluated using o-phenylenediamine (0.4 mg/mL) and 30% H_2_O_2_ in citrate–phosphate buffer (0.2 M sodium phosphate and 0.1 M citric acid). The plates were incubated for 10 min in the dark. The reaction was stopped by adding 3 M HCl, and absorbance at 490 nm was measured on a microtiter plate reader (Epoch, Bio Tek, Charlotte, VT, USA). The absorbance was converted to a percentage considering the value of the control sample *P. brasiliensis* that had not been treated with peptides as 100% adhesion. The difference between the absorbance of treated fungi relative to that of the control was defined as adhesion inhibition.

### 2.11. Therapeutic Use of Peptides in G. mellonella In Vivo Model of Infection

To evaluate the efficiency of the peptides in preventing and controlling *P. brasiliensis* infection, we treated the cells with peptides at 3 h before and after *P. brasiliensis* infection. Larvae groups (*n* = 10) were separately treated with 100 and 50 µg/larva of each peptide for 3 h before fungal infection with 5 × 10^6^ yeast cells/larva, as described by Scorzoni et al. [33], and with 50 µg/larva after fungal infection with the same inoculum size. The survival curve was evaluated daily for 7–8 days. As controls, the larvae were treated with PBS and infected with *P. brasiliensis*, whereas uninfected larvae were inoculated with PBS alone.

### 2.12. Effects of Peptides on Hemocyte Density and Phenoloxidase Activity

*Galleria mellonella* was treated with 50 and 100 µg/larvae of each peptide and incubated for 3 h at 37 °C for hemocyte density determination. The hemolymph was removed through a cut made with a scalpel and diluted 10-fold in cold anticoagulant solution (2% NaCl, 0.1 M glucose, 30 mM sodium citrate, 26 mM citric acid, and 10 mM ethylenediamine tetraacetic acid). Fluorescence-activated cell sorting (FACS) solution (200 µL) was added, and hemocyte counting was performed using flow cytometry in a FACSCanto (BD Biosciences, San Jose, CA, USA) as described by Scorzoni et al. [34].

The phenoloxidase activity after peptide treatment at 100 µg/larvae for 3 h was also evaluated as described by Scorzoni et al. [34]. The hemolymph was removed from five larvae and pooled in cold anticoagulant solution (50 µL hemolymph pool: 150 µL anticoagulant solution). The cells were separated by centrifugation at 27× *g* 5 min, and 50 µL of cell-free hemolymph samples was added to a 96-well microtiter plate containing 50 µL of PBS. As a positive control, 50 µL of 2.5 mg/mL of lipopolysaccharide was added rather than adding PBS; the plate was incubated at 25 °C for 5 min, and the substrate, 25 µL of 6 mM L-DOPA (Sigma), was added and incubated again for 1 h at 25 °C. Absorbance was measured at 490 nm.

### 2.13. Cell Wall Modifications Mediated by Exposure of Fungi to Peptides

Calcofluor white (CFW, Sigma) was used to evaluate the effects of peptide treatment on chitin levels in the *P. brasiliensis* cell wall using fluorescence microscopy. To evaluate glycosylated protein levels, we used concanavalin A-Alexa Fluor 488 (ConA-Alexa Fluor 488; Invitrogen) and evaluated the cells using flow cytometry.

For both assays, 10^6^ yeast cells/mL of *P. brasiliensis* in liquid BHI medium were incubated individually with each peptide at 400, 200, and 100 µg/mL (final concentration) for 3 h at 37 °C and 150 rpm.

To estimate chitin levels, the cells were centrifuged at 2655× *g* for 5 min, washed with PBS, and fixed in 100% methanol at −80 °C for 20 min and then at −20 °C for 20 min. The cells were rewashed, followed by incubation with 100 µg/mL of CFW in PBS for 30 min at 37 °C as described by de Curcio et al. [35]. The cells were washed and photographed with an INCell Analyzer 2000 (GE Healthcare, Little Chalfont, UK), and fluorescence intensity was analyzed using ImageJ 1.53e software (NIH, Bethesda, MD, USA). The corrected total cell fluorescence (CTCF) for CFW was determined as follows: CTCF = integrated density (area of yeast cells × mean background fluorescence). The integrated fluorescence density from the outline area was measured, and the background was determined around the perimeter of the yeast cells; a minimum of 100 cells were evaluated in triplicate under each condition, using a filter as described by Guttentag et al. [36].

For glycosylated protein analysis, yeast cells were washed after the peptide treatments described above and stained with 100 µg/mL of ConA-Alexa Fluor 488 at 37 °C, 150 rpm for 1 h. Fungal cells were washed, resuspended in PBS, and analyzed using a BD FACSCanto flow cytometer. The mean fluorescence intensity of 10,000 cells in the fluorescein isothiocyanate channels was determined [37].

### 2.14. Mycelium to Yeast Alterations in P. brasiliensis following Peptides Treatment

*Paracoccidioides brasiliensis* was cultured at 25 °C, 120 rpm, in liquid BHI medium supplemented with 1% glucose until reaching a complete transition to the mycelium phase (approximately 11 days, showing irregular, hyaline, septate, and ramified hyphae), as confirmed using optical microscopy. The culture was replaced with fresh BHI medium containing 50 and 100 µg/mL of each peptide separately, followed by incubation at 37 °C, 120 rpm. After complete transition of the untreated control to the yeast phase, as evaluated using microscopy, the cell morphology after all peptide treatments was analyzed as described by Nunes et al. [38], with morphology categorized into hyphae, differentiating hyphae, transforming yeasts, and yeasts.

### 2.15. Statistical Analysis

All statistical analyses were performed using GraphPad Prism version 5.0 software (GraphPad, Inc., La Jolla, CA, USA). Data were statistically analyzed using one-way analysis of variance (ANOVA) with a Bonferroni post hoc test. Survival curves were analyzed using a log-rank (Mantel–Cox) test. Statistical significance was set at *p* < 0.05.

## 3. Results

### 3.1. Enrichment of PbDrk1-Binding Phages and Identified Polypeptides

**Figure 1:**
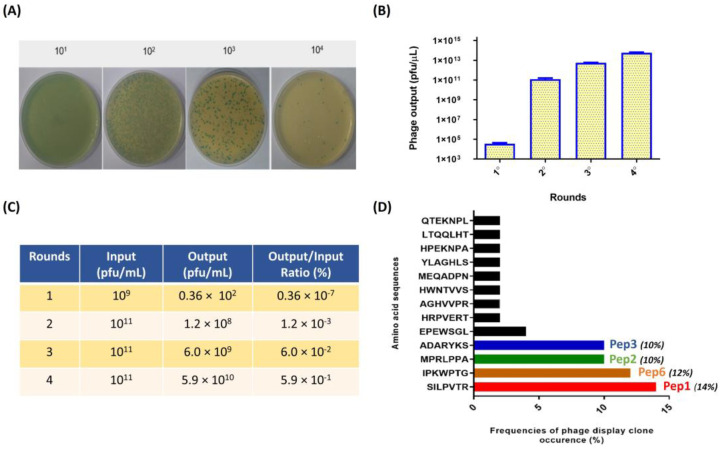
Enrichment of positive phage clones specifically binding to the recombinant PbDrk1 and most abundant peptides identified. (**A**) Example of counting plaques on LB plates with IPTG/X-gal media with serial dilution used to phage titering; recombinant phage from the phage library that carried the lacZα gene are shown as blue plaques on the plates. (**B**) Quantification of blue phage plaques recovered after each round of biopanning via M13KE used in Ph.D.TM—7 Phage Display Peptide Library on immobilized PbDrk1. (**C**) Enrichment of phages for each selection round from the phage-displayed heptapeptide library. (**D**) Amino acid sequences and frequencies of occurrence from 78 phage plaques randomly selected for sequencing after three affinity-selection rounds against PbDrk1. To simplify the naming of the peptides identified, the terms Pep1 (SILPVTR), Pep2 (MPRLPPA), Pep3 (ADARYKS), and Pep6 (IPKWPTG) were adopted. Pfu: plaque-forming unit.

Peptides bound to PbDrk1 protein were identified and characterized after four rounds of biopanning of recombinant PbDrk1 protein immobilized in 96-well plates. Phage output analysis was employed to evaluate the efficiency of phage recovery by counting the blue plaque-forming units (pfu) after titration in LB IPTG/X-Gal medium (Figure 1A). The amount of phage bound to PbDrk1 increased from 0.36 × 10^5^ pfu/µL in the first round to 5.9 × 10^13^ pfu/µL in the later round. Similar results were observed for enrichment, which were determined using the output/input ratio of phages after each round of biopanning (Figure 1B).

Following four rounds of biopanning, 78 single-phage plaques targeting PbDrk1 were selected separately for PCR screening and sequencing. DNA containing the nucleotide sequence encoding the peptide was amplified, purified, and sequenced. Using bioinformatics software, we identified the sequences encoding the 7-mer peptides and translated them into their corresponding amino acids. Several peptides were deduced and identified, and their frequencies are shown in Figure 1C as fractions relative to the total sequences. The most frequent peptides and their respective names designated in this study were SILPVTR (Pep1), IPKWPTG (Pep2), MPRLPPA (Pep3), and ADARYKS (Pep6), corresponding to 14%, 12%, 10%, and 10% of phage clones sequenced, respectively (Figure 1C). These peptides were then chemically synthesized with at least 95% purity, and 10 mg/mL stock solutions were prepared in PBS and stored at −20 °C until use.

Additionally, after the fourth round of biopanning, the eluted phages were titrated into a 96-well plate without PbDrk1, and no phage plaque was recovered, indicating the specificity of phage-target binding.

### 3.2. Binding of Polypeptides to PbDrk1 Protein and P. brasiliensis

**Figure 2:**
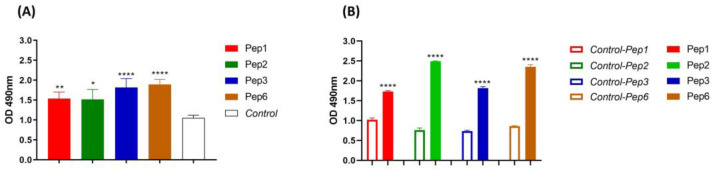
Peptides affinity to *P. brasiliensis* and PbDrk1 protein determined using ELISA. The figures show the ELISA absorbance values of peptides binding to (**A**) *P. brasiliensis* using anti-cell free and (**B**) PbDrk1 using an anti-Drk1 antibody as primary antibodies, respectively. In Control: peptides were omitted. In Control-Pep1, Control-Pep2, Control-Pep3, and Control-Pep6: PbDrk1 protein was omitted. **** *p* < 0.0001; ** *p* = 0.0061, and * *p* = 0.0158 compared to the respective controls using one-way ANOVA followed by Tukey’s multiple comparisons tests.

The reactivity of the peptides against PbDrk1 and yeast cells of *P. brasiliensis* was evaluated using ELISA, and 96-well plates without PbDrk1 or peptides were used as controls. As shown in Figure 2, all peptides significantly recognized both targets: *P. brasiliensis* yeast cells without significant differences among peptides according to the absorbance values (Figure 2A) and PbDrk1, with Pep1 and Pep3 showing reduced absorbance compared to those of the other peptides (Figure 2B).

### 3.3. Antifungal Activity of Synthetic Polypeptide and Its Potentiation of Antifungals

None of the peptides evaluated showed antifungal activity against *P. brasiliensis* at concentrations of 0.765–1.600 µg/mL. Moreover, when used with AMB and Itra, the peptides potentiated the antifungal effects of these well-established drugs. Once the peptides showed no antifungal activity, their concentration was fixed (200 µg/mL) and varied from those of AMB and Itra. All peptides enhanced the growth inhibition of *P. brasiliensis* at 0.25 µg/mL AMB (AMB concentration at which, when used alone, did not reduce *P. brasiliensis* growth, showing 100% growth).

**Figure 3:**
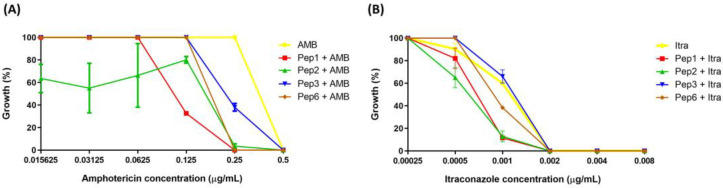
Potentiation effect of selected peptides on antifungal activity. The peptide concentrations were fixed at 200 µg/mL, and antifungal concentrations ranged from (**A**) 0.015625 to 0.5 µg/mL to amphotericin (AMB) and (**B**) 0.00025 to 0.008 to itraconazole (Itra). *p* < 0.0001 for all peptides with 0.25 µg/mL of AMB; *p* = 0.0001 (to Pep1), *p* = 0.0138 (to Pep2) and *p* = 0.0022 (to Pep6) with 0.001 µg/mL of Itra analyzed using two-one way ANOVA with Dunnett’s comparison test.

The peptides Pep1, Pep2, and Pep6 showed 100% growth inhibition and Pep3 showed 62% growth inhibition (Figure 3A), suggesting that the peptides potentiated the effects of the drugs when one drug or compound did not elicit a response on its own but increased the response to another drug.

Pep1, Pep2, and Pep6 increased the growth inhibition of *P. brasiliensis* when an Itra concentration of 0.001 µg/mL was used. The antifungal alone inhibited 40% of fungal growth. The peptide–Itra association led inhibited growth by 88.4%, 87.2%, and 60.6% for Pep1, Pep2, and Pep6, respectively, contributing to the antifungal action. No differences were observed when Pep3 was used (Figure 3B).

### 3.4. Cytotoxicity of Polypeptides in Pneumocytes A549 Cells and G. mellonella

**Figure 4:**
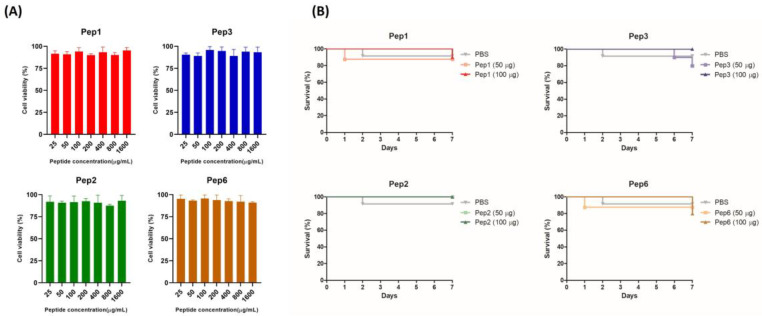
Toxicity of selected peptides for pneumocytes A549 cells and *Galleria mellonella*. (**A**) Cytotoxicity in A549 cells determined using the resazurin method. Monolayer cells were treated with increasing concentrations of indicated peptides ranging from 25 to 1.600 µg/mL; after 24 h, metabolic activity was quantified using the resazurin assay, and the absorbance values were normalized to those of the untreated control. The result was expressed as cell viability (%) and analyzed using one-way ANOVA with Tukey’s multiple comparison test. (**B**) Toxicity in *G. mellonella* invertebrate model; groups of larvae (n = 10) were injected with 50 and 100 µg/larva of indicated peptides; survival was monitored for 7 days post-injection and analyzed using the log-rank (Mantel–Cox) test. *p* = 0.9447 (to Pep1), *p* = 0.4724 (to Pep2), *p* = 0.3683 (to Pep3), and *p* = 0.7828 (to Pep6).

The toxicity of the peptides was evaluated in vitro and in vivo using A549 and *G. mellonella* models. The mitochondrial respiratory activity of A549 cells was assessed using the resazurin assay. The peptides showed no cytotoxicity in concentrations of 25–1.600 µg/mL, with the percentage of viable cells close to or greater than 90% (Figure 4) (below 30% is acceptable cytotoxicity according to ISO 10993-5, 2009). Peptide treatment with 50 and 100 µg/larva concentrations did not have toxic effects on *G. mellonella*, with larvae survival rates of close to or greater than 80% (Figure 4B).

### 3.5. Some Peptides against PbDrk1 Inhibited Adhesion Abilities to A549 Cells but Not to ECM

**Figure 5:**
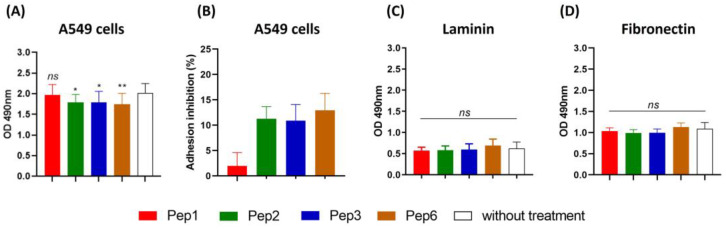
Adhesion inhibition to A549 cells and extracellular matrix mediated by peptides. *Paracoccidioides brasiliensis* yeast cells were treated for 1 h with 200 µg/mL of each peptide and then used in a monolayer of A549 cells and 96-well coated plate with 10 µg/mL of each laminin and fibronectin for 15 h of incubation and evaluated using ELISA. (**A**) Absorbance at 490 nm following ELISA for A549 cells, and (**B**) adhesion inhibition to A549 cells as a percentage for each peptide. (**C**) ELISA for laminin. (**D**) ELISA for fibronectin. *ns*: not significant, * *p* < 0.05 and ** *p* < 0.01. *p* = 0.0156 (to Pep2; 11.2% reduction), *p* = 0.0178 (to Pep3, 10.9% reduction), *p* = 0.0063 (to Pep6, 12.9% reduction) compared to the control without treatment. One-way ANOVA followed by Dunnett’s multiple comparison test.

Treatment of *P. brasiliensis* with Pep2, Pep3, and Pep6 peptides reduced the ability of yeast cells to adhere to A549 cells, with inhibition rates of 11.2%, 10.9%, and 12.9%, respectively, suggesting that the peptides bind to *P. brasiliensis* and alter their interactions with host cells (Figure 5A,B). No differences were observed in *P. brasiliensis* binding to laminin or fibronectin after peptide treatment (Figure 5C,D).

### 3.6. Paracoccidioides Brasiliensis-Cell Wall Modifications Mediated by Identified Peptides

**Figure 6:**
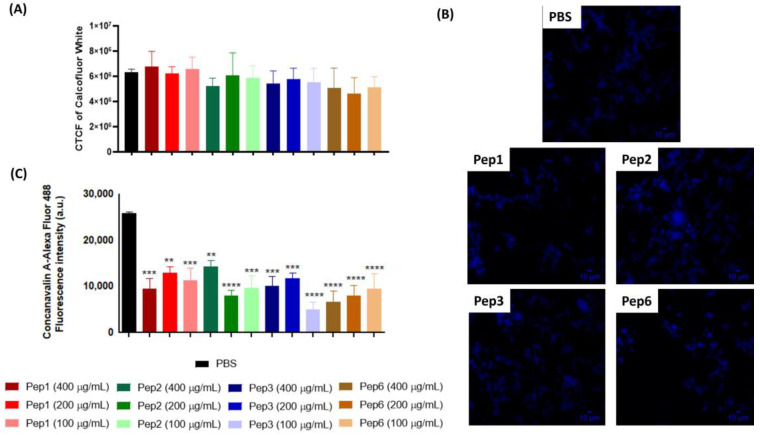
Alterations of cell-wall components of *P. brasiliensis* after peptide treatment. Yeast cells of *P. brasiliensis* were treated for 3 h with 100, 200, and 400 µg/mL of each peptide. The cells were labeled with (**A**,**B**) calcofluor white (CFW) to measure chitin levels using fluorescence microscopy and (**C**) concanavalin-Alexa Fluor conjugated to glycosylated proteins levels using cytometry. ** *p* < 0.01, *** *p* < 0.001, **** *p* < 0.0001 compared to the PBS control group using one-way ANOVA followed by Dunnett’s multiple comparisons tests. CTCF: corrected total cell fluorescence. Scale bars are included = 10 µM.

Chitin levels in *P. brasiliensis* after treatment with all peptides were determined using CFW, a fluorescent dye that specifically binds to chitin. The CTCF of CFW of *P. brasiliensis* did not change after peptide treatment compared to that of the control and showed no differences among concentrations (Figure 6A,B). Moreover, the protein glycosylation profile after peptide treatment, as determined by staining with ConA-Alexa Fluor 488, was significantly reduced for all peptides and concentrations compared to that of the control (Figure 6C).

### 3.7. Mycelium to Yeast Transition of P. brasiliensis Is Altered by Contact with Peptides

**Figure 7:**
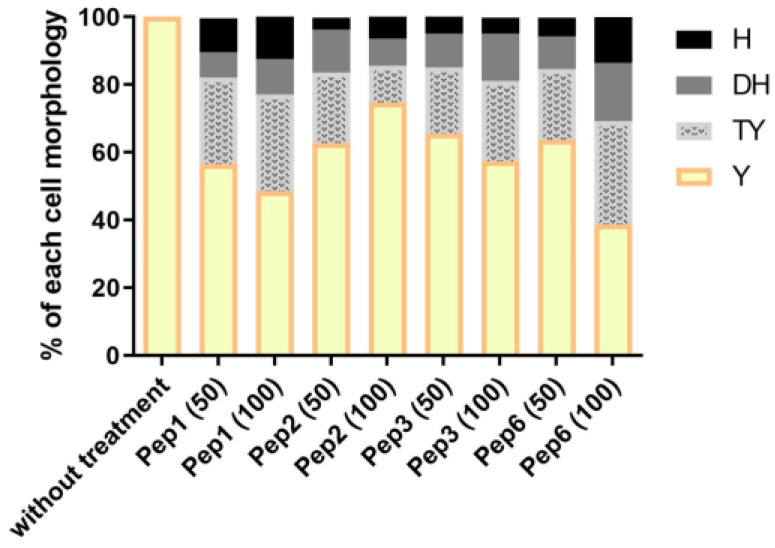
Morphological transition in *P. brasiliensis* after peptide treatment. The mycelium phase of *P. brasiliensis* was treated with different concentrations (50 and 100 µg/mL) of each peptide and incubated at 37 °C until the control (without treatment) reached complete conversion to the yeast phase (9 days). Aliquots of each group were analyzed using optical microscopy to discriminate the cell morphology as described by Nunes et al. [38]; H: hyphae, DH: differentiate hyphae, TY: transforming yeasts, and Y: yeasts.

Mycelial treatment with all peptides altered the ability of *P. brasiliensis* to enter the parasitic phase, as shown in Figure 7. After 9 days, when the control (without treatment) showed 100% of cells in the yeast phase, the other conditions presented varying levels of other morphological stages, indicating that peptide treatment delays the dimorphism process.

### 3.8. Prophylactic and Therapeutic Use of Drk1-Binding Peptides in G. mellonella Model of Infection

**Figure 8:**
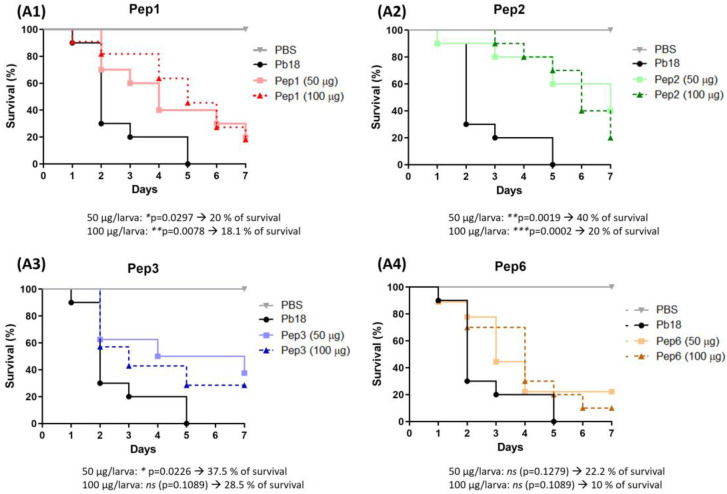
Use of peptides to protect *G. mellonella* larvae against *P. brasiliensis* infection. The survival curve of *G. mellonella* larvae (n = 10 per group) was treated with the selected peptides (**A1**) to Pep1, (**A2**) to Pep2, (**A3**) to Pep3 and (**A4**) to Pep6 at both concentrations (50 and 100 µg/larva) 3 h before infection with *P. brasiliensis*. Statistical analyses were performed using the log-rank (Mantel–Cox) test of three independent experiments. * *p* < 0.05, ** *p* < 0.01, *** *p* < 0.001 and *ns:* not significant. The *p* values for each peptide and concentration as well as the % of larvae survival are described in the figure.

Peptide efficacy was evaluated in vivo in a *G. mellonella* infection model. Treatment before and after infection with *P. brasiliensis* resulted in remarkable larval survival. The peptides showed variations in the survival rate without significant differences among the evaluated concentrations of each peptide. The most effective treatments were those performed with peptides 1, 2, and 3, which rescued the larvae. In 7 days, the survival rates were 20% and 18.1% for Pep1 and 40% and 20% for Pep2 to 50 µg and 100 µg/larva, respectively, and 37.5% to Pep3 to 50 µg/larva, when compared to infected larvae that were not treated with peptides. All larvae were dead after 7 days. Although the curves for Pep3 at 100 µg/larva and for Pep6 did not significantly differ between the two administered concentrations, all conditions delayed larval death (Figure 8).

**Figure 9:**
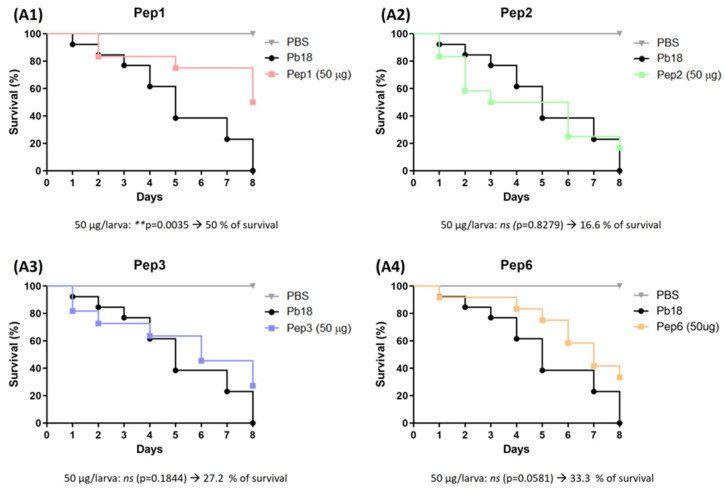
Use of peptides to combat *G. mellonella* infection with *P. brasiliensis.* Survival curve of *G. mellonella* larvae (*n* = 10 per group) infected with *P. brasiliensis* and treated with the selected peptides (**A1**) to Pep1, (**A2**) to Pep2, (**A3**) to Pep3 and (**A4**) to Pep6 at 50 µg/larva for 3 h. Statistical analyses were performed using the log-rank (Mantel–Cox) test of three independent experiments. ** *p* < 0.01, and *ns:* not significant. The *p* values for each peptide and concentration as well as the % of larvae survival are described in the figure.

After detecting no differences with the peptide dose, we administered 50 µg peptide/larva after *P. brasiliensis* infection. A significant increase in survival was observed in the sample treated with Pep1 (50% survival) compared to that of the untreated group, which died at the end of 8 days. Although the survival curves of Pep2, Pep3, and Pep6 did not significantly differ, survival was increased by the end of the experiment (Figure 9).

### 3.9. Effects of Peptides in Hemocyte Density and Phenoloxidase Activity of G. mellonella

**Figure 10:**
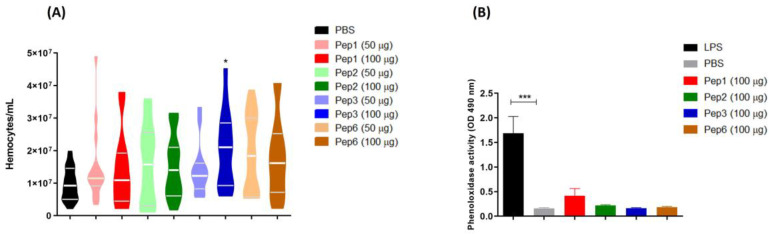
*Galleria mellonella* immunomodulation following peptide treatment. Larvae (n = 10) of *G. mellonella* were treated with each peptide at different concentrations (50 and 100 µg/larva for hemocyte assay and 100 µg/larva for phenoloxidase assay) for 3 h, and the (**A**) hemocytes density and (**B**) phenoloxidase activity were evaluated. * *p* = 0.0331 compared to PBS control, analyzed using the Kruskal–Wallis test followed by Dunn’s multiple comparison test. *** *p* < 0.0001 compared to PBS control with one-way ANOVA with Tukey’s multiple comparison test.

The effect of peptide treatment on the immune response by increasing the hemocyte concentration was evaluated. All peptides increased the hemocyte density but significantly differed from the effects of Pep3 at 100 µg/larva (Figure 10A). Additionally, peptide treatment did not induce the production of phenoloxidase, an enzyme involved in the humoral response in *G. mellonella* (Figure 10B).

## 4. Discussion

Despite the availability of antifungal agents, limited progress has been made in the treatment of PCM, a neglected mycosis that poses economic and social challenges. Conventional therapy is associated with toxicity and has an extended duration, spanning months to years. This has prompted the search for molecules with greater effects [12], which requires a detailed understanding of the pathobiology. However, fewer studies have focused on *P. brasiliensis* than on other fungi such as *Candida* spp. and *Cryptococcus* spp. [4].

For antifungal development, diverse avenues can be explored, including drug repurposing, creation of new drugs from natural or synthetic sources, synthesis, antifungal evaluation, and utilization of nanotechnology for drug delivery [1]. Phage display technology can reveal peptides that act against biological and nonbiological targets for identifying proteins or peptide drugs, ligand identification, vaccine development, and antibody engineering [39].

Drk1 is a suitable candidate for new therapy development because of its potential involvement in fungal virulence [14,17,18,20] and absence from mammalian cells, thereby minimizing the risk of host toxicity [40,41]. A phage library displaying random 7-mer peptides was screened against immobilized PbDrk1 recombinant protein. This peptide library was added to the phage surface, followed by biopanning. Following phage enrichment, the genome was sequenced. A bioinformatics approach was employed to analyze the sequences, leading to identification of the peptide sequence.

After four enrichment and amplification rounds, the 7-mer library was enriched in various peptides. A total of 78 phage clones were used to confirm the displayed peptide sequences. The most frequently recurring peptides (>10%) were selected and synthesized for subsequent tests.

Analysis of the targeting ability of the peptides against PbDrk1 and *P. brasiliensis* yeast cells demonstrated that all peptides bound to both targets at comparable levels. However, we did not evaluate the specific binding site, which may explain some of the differences observed among the selected peptides and their inherent characteristics.

The fungicide fludioxonil, a phenylpyrrole used as an agrochemical, may induce hyperactivation of the HOG signaling pathway via group III HHK [42,43,44]. Thus, we investigated whether the peptides could inhibit the growth of *P. brasiliensis*. Our results revealed that none of the peptides inhibited bacterial growth. Notably, such comparisons are not straightforward because fludioxonil does not directly interact with Drk1. Moreover, the mechanism of action of fludioxonil may be more complex than is currently understood. We hypothesized that fludioxonil induces the oxidative stress sensed by HHK, leading to hyperactivation of the HOG pathway and cell death. However, cells subjected to oxidative stress remained viable. This result suggests that oxidative stress triggered by fludioxonil alone is insufficient to explain the mechanism of cell death. Subsequent analysis revealed a connection between fludioxonil and aldehyde stress. Fludioxonil treatment led to a surge in the levels of the aldehyde methylglyoxal, a highly reactive compound that causes cellular damage. Methylglyoxal can be produced in small amounts when cells break down sugar molecules. These cells typically produce enzymes capable of degrading aldehydes. Fludioxonil inhibits these enzymes, resulting in excess aldehyde methylglyoxal. This elevated aldehyde content triggers HHK activation through sulfur amino acids, which are sensitive to aldehyde groups. Triosephosphate isomerase, one of the targets of fludioxonil, contributes to increased levels of aldehyde methylglyoxal. This, in turn, alters HHK activity from kinase to phosphatase, affecting downstream elements of the HOG pathway, such as Ypd1, and ultimately culminating in HOG pathway activation [45].

Although the respective peptides did not exhibit standalone antifungal activity, we showed their potential to increase the activity of two conventional antifungals employed in PCM treatment when combined with these: Itra and AMB. Additionally, mutants of genes related to the MAPK pathway displayed increased susceptibility to both drugs [46]. Furthermore, knockdown of DRK1 in *P. brasiliensis* conferred increased susceptibility to Itra in yeast cells [18]. These findings provide insight into potential strategies for enhancing antifungal efficacy through synergistic combinations and targeted genetic modifications.

Various mechanisms of action have been described for both drugs, suggesting their ability to target the cellular antioxidant system and categorizing them as oxidative stress drugs [46,47,48,49,50,51]. Fungi activate a response that integrates MAPK pathways, including the HOG MAPK system, to counteract oxidative stress [52,53]. In the present study, all peptides targeting PbDrk1 exhibited an enhanced effect in combination with AMB against *P. brasiliensis* in vitro, in the following order of inhibition capacity: Pep1 = Pep2 = Pe6 > Pep3. Similarly, Itra increased growth inhibition when combined with peptides in the order of Pep1 = Pep2 > Pep6. This result suggests that these peptides can bolster the antifungal responses and that Drk1 in *P. brasiliensis* is involved in sensing oxidative stress.

The peptides showed no toxic effects toward either *G. mellonella* or A549 cells at the concentrations evaluated. Previous research demonstrated the presence of Drk1 on the fungal surface or as a transmembrane protein, among other sites, using an anti-PbDrk1 antibody. Moreover, treatment of yeast cells with the same antibody reduces the ability of the fungus to interact with host cells and attenuates the virulence of *G. mellonella* [18]. Hence, we explored whether the peptides exerted similar inhibitory effects on adhesion to pneumocytes and two types of ECM. All peptides except Pep1 reduced the capacity of the fungus to adhere to pneumocytes. However, no reduction was observed in fungal binding to laminin and fibronectin, suggesting that the infection condition of pneumocytes presents a context where *P. brasiliensis* encounters altered environmental conditions, and the peptide interaction with PbDrk1 results in a reduced fungal capacity to recognize and respond to this specific condition. This effect was not evident in the evaluated ECM. The host microenvironment is in constant flux, in which the fungus must adapt and thrive [54]. The literature contains reports on the involvement of a two-component system and its downstream pathway, Hog1, in fungal signal transduction, which orchestrates responses to various stresses such as osmotic and oxidative stress, cell wall synthesis, virulence, cell adhesion, and morphogenesis [55,56,57,58,59,60,61].

Although treatment with the peptides did not affect the chitin content of *P. brasiliensis*, it did reduce the levels of glycosylated proteins. ConA, a lectin with affinity for glycolipids and glycoproteins, including those on the fungal cell surface, was used to visualize these sugar structures. All peptide treatments reduced ConA fluorescence, indicating lower levels of sugar moieties on lipids and proteins on the fungal surface. These altered glycosylation patterns caused by peptide treatment may affect the fungal physiology, pathogenesis, and host interactions. The consequences may include loss of cell wall integrity, reduced adherence to host tissues or other surfaces, altered recognition by immune cells and molecules, increased susceptibility to antifungals/stresses, attenuation of virulence, and changes in fungal signaling and communication.

*Galleria mellonella* larvae were treated with the peptides and infected with *P. brasiliensis*. A parallel experiment was performed in which the larvae were first treated and then infected. This dual approach was used to explore the potential immune system modulation induced by these peptides and assess their efficacy in combating PCM. Both doses (50 and 100 µg/larva) of Pep1 and Pep2, along with 50 µg/larva of Pep3, administered before infection, significantly increased larval survival compared to that of the untreated group. Notably, there were no significant differences between the effects of different peptide concentrations. However, Pep3 at 100 µg/larva and Pep6 at both concentrations delayed larval death and led to increased survival by the end of the experiment. Regarding treatment after infection, only Pep1 exhibited a significant effect, with a 50% survival rate at the end of the assay. Although other peptides also enhanced survival, the differences were not significant. Using kinase inhibitors makes it challenging to select the appropriate drug concentration because cellular ATP availability can vary widely [62,63,64]. This notable efficacy of Pep1 may be attributed to its polar nature, as assessed using the ProtParam tool (http://web.expasy.org/protparam; accessed on 1 December 2022; Supplementary Data, Table S1). This polarity may hinder its distribution within the *G. mellonella* fat body, allowing it to remain in the hemolymph for an extended period and increasing the contact time with the fungus post-infection.

The peptides did not affect phenol oxidase activity. Phenoloxidase catalyzes the formation of melanin, which encapsulates the infection site. Additionally, the peptides did not alter hemolymph coagulation, opsonization, or hemocyte density, which influence phagocytic activity [65,66,67,68]. The immune response in this infection model involves additional factors, including antimicrobial peptides (galiomycin and gallerimycin) and nitric oxide production by hemocytes [69,70]. These factors may contribute to the efficacy of the peptides in preventing infection or their ability to act directly on *P. brasiliensis* yeast cells within 3 h of pretreatment.

Only Pep1 significantly enhanced post-infection survival, achieving a 50% survival rate at the end of the assay. Although the other peptides also increased survival, the differences were not significant. These results highlight the potential value and depth of its application in murine models.

Given the correlation between Drk1 and *P. brasiliensis*’s dimorphism and yeast morphology maintenance [18,20], we further investigated the effects of peptide binding to this target on the mycelium-to-yeast transition, which is an essential aspect of virulence and pathogenesis [71,72]. Remarkably, all peptides decreased *P. brasiliensis*’s ability to transition from the mycelium to the yeast form at concentrations that did not compromise fungal viability. These findings highlight that peptides designed to bind Drk1 significantly alter the dimorphism of this pathogen.

## 5. Conclusions

We isolated ligands targeting Drk1 of *P. brasiliensis*, which is absent from mammalian cells. We identified several peptides, with four emerging as the most frequently recognized peptides. By strategically targeting Drk1, these peptides exhibited a remarkable ability to inhibit *P. brasiliensis* from transitioning between mycelium and yeast forms, which is a pivotal phase in the pathogenic life cycle of the organism.

Although they lack direct antifungal activity, these peptides can potentiate the effects of certain conventional antifungals and, thus, may reduce the required doses of antifungal agents. This strategic augmentation may alleviate the toxicity associated with PCM treatment, which warrants further exploration.

Importantly, the peptides showed multifaceted effects on fungal–host cell adhesion. By attenuating this critical interaction, peptides can cause under-glycosylation in yeast cells. This phenomenon supports their potential utility against *P. brasiliensis* infections in a *G. mellonella* model, adding another layer of efficacy to their repertoire.

Our further studies will focus on characterizing the intricate binding interfaces between these peptides and Drk1. Additionally, we aim to determine the complex cell signaling dynamics and provide insight into the intricate mechanisms underpinning the effects of peptides. These findings improve the understanding of *P. brasiliensis* pathogenesis and provide a foundation for the clinical translation of novel therapeutic strategies.

## Data Availability

The raw data supporting the conclusions in this article will be made available by the authors, without undue reservations, but all relevant data are within the paper.

## Supplementary Materials

The following supporting information can be downloaded at https://www.mdpi.com/article/10.3390/jof9100980/s1: Table S1: Amino acid sequences and characteristics of the peptides calculated by ExPASy tool ProtParam.
