# Supplementary material for: Polypeptides Targeting Paracoccidioides brasiliensis Drk1
Source: J Fungi (Basel). 2023 Sep 29;9(10):980. doi: 10.3390/jof9100980 (PMC10607314; doi:10.3390/jof9100980)
Supplement: Supplementary file 1 [file jof-09-00980-s001.zip › jof-2430471-supplementary.pdf]

Table S1: Amino acid sequences and characteristics of the peptides calculated by ExPASy tool ProtParam.

Peptide	*Sequence*	**Net Charge**	**Weights g/mol**	**PI**	**(+)R**	**(-)R**	**II**	**AI**	**Gravy value**
**Pep1**	SILPVTR	1	784.95	9.47	1	0	78.99	152.86	0.700
**Pep2**	MPRLPPA	1	780.98	9.50	1	0	143.11	70.00	-0.257
**Pep3**	ADARYKS	1	809.88	8.63	2	1	-14.33	28.57	-1.486
**Pep6**	IPKWPTG	1	797.55	8.75	1	0	-7.67	55.71	-0,657

PI: isoelectric point; **(+)R:** total number of positively charged residues; **(-)R:** total number of negatively charged residues; **II:** Instability index; **AI:** aliphatic index; **Gravy:** grand average of hydropathicity.
